# Flow Cytometry Method as a Diagnostic Tool for Pleural Fluid Involvement in a Patient with Multiple Myeloma

**DOI:** 10.4084/MJHID.2012.063

**Published:** 2012-10-03

**Authors:** Muzaffer Keklik, Serdar Sivgin, Cigdem Pala, Celalettin Eroglu, Gulsah Akyol, Leylagul Kaynar, M. Yavuz Koker, Demet Camlica, Ali Unal, Mustafa Cetin, Bulent Eser

**Affiliations:** 1 Erciyes Stem Cell Transplantation Hospital, Department of Hematology, Faculty of Medicine, Erciyes University, Kayseri, Turkey; 2 Department of Radiation Oncology, Faculty of Medicine, Erciyes University, Kayseri, Turkey; 3 Flow Cytometry Unit, Department of Hematology, Faculty of Medicine, Erciyes University, Kayseri, Turkey; 4 Flow Cytometry Unit, Faculty of Medicine, Erciyes University, Kayseri, Turkey

## Abstract

Multiple myeloma is a malignant proliferation of plasma cells that mainly affects bone marrow. Pleural effusions secondary to pleural myelomatous involvement have rarely been reported in the literature. As it is rarely detected, we aimed to report a case in which pleural effusion of a multiple myeloma was confirmed as true myelomatous involvement by flow cytometry method. A 52-years old man presented to our clinic with chest and back pain lasting for 3 months. On the chest radiography, pleural fluid was detected in left hemithorax. Pleural fluid flow cytometry was performed. In the flow cytometry, CD56, CD38 and CD138 found to be positive, while CD19 was negative. True myelomatous pleural effusions are very uncommon, with fewer than 100 cases reported worldwide. Flow cytometry is a potentially useful and simple method for detection of pleural fluid involvement in multiple myeloma.

## Introduction

Multiple myeloma is malign proliferation of plasma cells and it mainly affects bone marrow; however, it may affect thorax as skeletal abnormalities, plasmocytomas, pulmonary infiltrates and pleural effusion^1^. Pleural effusion may be myelomatous or non-myelomatous origin. It has been reported that pleural effusion might develop in 6% of the patients due to various reasons including congestive heart failure due to amyloidosis, chronic renal failure, nephritic syndrome, hypoalbuminemia, pulmonary emboli, secondary neoplasm or infection.^2^,^3^ However, myelomatosis pleural effusion is rarely seen.^4^ Recently, flow cytometry has increasingly become important in the diagnosis, prognostication and follow-up of multiple myeloma. Immunophenotypic studies of Multiple Myeloma patients have been performed for more than 20 years.^5^ This method enables to diagnose with 6 different colour staining system using surface antibodies of any cell and does not require high cost. Concerning plasma cell dyscrasias, it has been shown that, based on the expression of several markers, normal and myelomatous plasma cells can be easily differentiated. The antigens most frequently used for the identification of aberrant plasma cell phenotype include CD19, CD45, and CD56 in combination with CD38/CD138.^6^,^7^ In flow cytometric evaluation; CD19 negativity is considered as a diagnostic criterion of multiple myeloma and distinguishes MM from lymphomas. Also we analyze presence of CD56 expression for criteria of malign potency. It is a general consideration that CD38 and CD138 positivity should be analyzed for establishing the diagnosis of MM. Whether or not normal plasma cells are phenotypically different from myelomatous plasma cell remains controversial although some antigenic combinations such as CD19−/CD56++ could probably help to identify the malignant nature of plasma cell.^7^,^8^ In our case; CD56, CD38 and CD138 expressions were found positive.

As it is rarely detected, we aimed to report a case in which pleural effusion of a multiple myeloma was confirmed as true myelomatous involvement by flow cytometry method.

## Case

**Figure 1:**
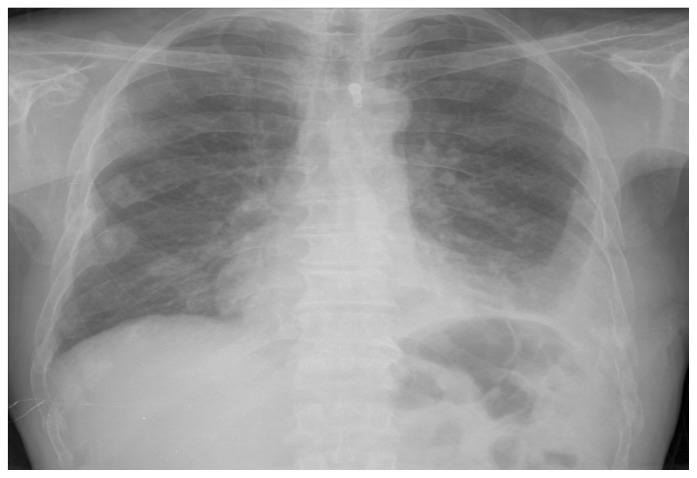
Chest radiograph showing pleural effusion on the left

A 52-years old man presented to our clinic with chest and back pain lasting for 3 months. On the chest radiography, pleural fluid was detected in left hemithorax (Figure 1). On thorax CT, it was also detected that there were lytic bone lesions at level of 11^th^ and 12^th^ ribs and pleural thickening at paravertebral site on the left. In laboratory evaluations, following findings were observed: hemoglobin: 7.1g/dl.(14–18), white blood cells:11.26 × 10^3^/μL(4.8–10.8), platelets: 787 × 10^3^/μL., creatinine: 3.1mg/dl.(0.6–1.1).Uric acid: 6.1 mg/dl (2.6–6), calcium: 14.7mg/dL (8.8–10.6), total protein:10.7g/dL (6.4–8.3), albumin: 1.8g/dL (3.5–5.2). Serum protein electrophoresis revealed a hypoalbuminemia (21.1%; range; 55.8–66.1) associated with an increase in γ globulins (42.1%; range; 11.1–18.8). IgG-Kappa monoclonal paraproteinemia was detected in immunofixation tests (IgG: 4460mg/dL. reference interval, 850–1330; Kappa: 1640mg/dL, reference interval, 630–1350). ß-2 microglobulin was found as 2.71 mg/dlL (reference interval: 1.42–3.21). Bence-Jones protein was found to be negative in 24-hours urine collection. A thoracentesis was performed, which revealed serofibrinous fluid with a protein level of 6.3gr/dl and white blood cell count of 6.65 × 10^3^μL consisting of lymphocytes (80%) and neutrophils (20%).

Bacterial and micobacterial culture tests of pleural fluid were reported as negative. Biopsy was performed on the mass at rib, which was reported as CD138(+), CD20(−) plasmocytoma. Pleural fluid flow cytometry was performed by using FACSCalibur flow cytometer (Becton-Dickinson, Erembodegem, Belgium). In the flow cytometry, CD56, CD38 and CD138 found to be positive, while CD19 was negative. Bone marrow biopsy was reported as CD38 (+) and CD20(−) atypical plasma cell infiltration. T(4;14), 17p13,1 (p53 gene) and 13q14,3(Rb gene) was found as negative by FISH method.

The patient was scheduled for VAD (vincristine, doxorubicin, dexamethasone) chemotherapy, biphosphonate therapy and involved-field radiotherapy.

## Discussion

Multiple Myeloma is a clonal late B-cell disorder in which malignant plasma cells expand and accumulate in the bone marrow, leading to cytopenias, bone resorption and the production (in most cases) of the characteristic monoclonal protein.^9^ Areas other than bone marrow may be a marker of thoracic involvement which affects about 6% of patients with Multiple Myeloma.^3^,^10^ Pleural effusions secondary to pleural myelomatous involvement have rarely been reported in the literature. True myelomatous pleural effusions are very uncommon, with fewer than 100 cases reported worldwide.^11^,^12^ The most common causes of pleural effusion associated with Multiple Myeloma are heart failure, renal failure, effusions related to pneumonia and amyloidosis.^13^ Recently, flow cytometry has gained increasing importance in the diagnosis, and prognostication of multiple myeloma. Flow cytometry is a potentially useful diagnostic tool for clinical practice. Advantages of flow cytometry include its ability to distinguish between normal, reactive and malignant plasma cells.^14^,^15^ In addition, it can be used in the evaluation of body fluids such as pleural fluid.

Myelomatous effusions are rarely seen, in which demonstration of monoclonal protein and atypical plasma cells on pleural fluid electrophoresis and histological diagnosis by pleural biopsy is used as diagnostic procedures. Pleural involvement can be diagnosed by presence of plasma cells in pleural fluid or pleural biopsy. Large persistent pleural effusions refractory to diuretics and thoracentesis are more likely to be due to pleural amyloid infiltration. Agarwall et al. demonstrated that in their case; the pleural fluid cytology did not reveal any myelomatous cell, and the recurrent effusions were secondary to pulmonary amyloidosis.^16^ In our case, plasmocytoma diagnosis was confirmed by biopsy from the mass on ribs, whereas pleural involvement of multiple myeloma was detected by using flow cytometry of pleural fluid.

In conclusion, we presented our case; as it has been rarely reported, although flow cytometer is a simple method for detection of pleural fluid involvement in Multiple Myeloma.

## Figures and Tables

**Figure 2:**
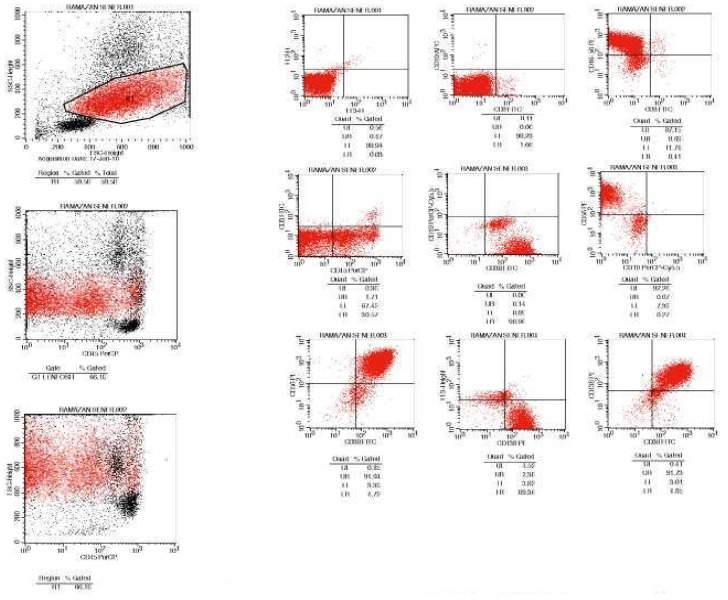
Flow cytometer analysis is consistent with involvement of pleural fluid by multiple myeloma with CD56, CD38 and CD138 positivity.
